# The changes of blood-based inflammatory biomarkers after non-pharmacologic interventions for chronic low back pain: a systematic review

**DOI:** 10.1186/s12891-024-07289-1

**Published:** 2024-03-08

**Authors:** Laura Maria Puerto Valencia, Yangyang He, Pia-Maria Wippert

**Affiliations:** 1 Medical Sociology and Psychobiology, University of Potsdam, Potsdam, Germany; 2 Faculty of Health Sciences Brandenburg [joint Faculty, Brandenburg Medical School Theodor Fontane, University of Potsdam, Brandenburg University of Technology Cottbus – Senftenberg], Brandenburg, Germany

**Keywords:** Anti-inflammatory cytokines, Interleukin, Pain management, Pro-inflammatory cytokines, Tumor necrosis factor alpha

## Abstract

**Background:**

Chronic low back pain (CLBP) is a prevalent and debilitating condition, leading to significant challenges to both patients and the governmental healthcare system. Non-pharmacologic interventions have received increasing attention as potential strategies to alleviate chronic low back pain and improve patient outcomes. The aim of this systematic review was to comprehensively assess the changes in blood inflammatory biomarkers after non-pharmacologic interventions for CLBP patients, thus trying to understand the complex interactions between non-pharmacologic interventions and inflammatory biomarker changes in CLBP.

**Methods:**

A thorough search (from January 1st, 2002 to October 5th, 2022) of PubMed, Medline (platform Web of Science), and the Cochrane Library (platform Wiley Online Library) were conducted, and inclusion criteria as well as exclusion criteria were refined to selection of the studies. Rigorous assessments of study quality were performed using RoB 2 from Cochrane or an adaptation of the Downs and Black checklist. Data synthesis includes alterations in inflammatory biomarkers after various non-pharmacologic interventions, including exercise, acupressure, neuro-emotional technique, and other modalities.

**Results:**

Thirteen primary studies were included in this systematic review, eight randomized controlled trials, one quasi-randomized trial, and four before-after studies. The interventions studied consisted of osteopathic manual treatment (one study), spinal manipulative therapy (SMT) (three studies), exercise (two studies), yoga (two studies) and acupressure (two studies), neuro-emotional technique (one study), mindfulness-based (one study) and balneotherapy study (one study). Four studies reported some changes in the inflammatory biomarkers compared to the control group. Decreased tumor necrosis factor-alpha (TNF-α) after osteopathic manual treatment (OMT), neuro-emotional technique (NET), and yoga. Decreased interleukin (IL)-1, IL-6, IL-10, and c-reactive protein (CRP) after NET, and increased IL-4 after acupressure. Another five studies found changes in inflammatory biomarkers through pre- and post-intervention comparisons, indicating improvement outcomes after intervention. Increased IL-10 after balneotherapy; decreased TNF-α, IL-1β, IL-8, Interferon-gamma, interferon-γ-induced protein 10-γ-induced protein 10 after exercise; decreased IL-6 after exercise and SMT; decreased CRP and chemokine ligand 3 after SMT.

**Conclusion:**

Results suggest a moderation of inflammatory biomarkers due to different non-pharmacologic interventions for CLBP, generally resulting in decreased pro-inflammatory markers such as TNF-α and IL-6 as well as increased anti-inflammatory markers such as IL-4, thus revealing the inhibition of inflammatory processes by different non-pharmacologic interventions. However, a limited number of high-quality studies evaluating similar interventions and similar biomarkers limits the conclusion of this review.

**Supplementary Information:**

The online version contains supplementary material available at 10.1186/s12891-024-07289-1.

## Introduction

Chronic low back pain (CLBP) is a widespread condition, with a prevalence in Germany adults by 2020 of 37% (point-prevalence), 76% (1-year prevalence), and 16% lifetime [1]. Very often low back pain patients have a “non-specific” disease, so a specific cause of the pain is missing (90% frequently estimated) [2, 3]. For only up to 15% of patients, a pathology diagnosis is defined [4]. The significant impact of CLBP on life quality and social economy, especially due to loss of productivity, needs effective management strategies.

Non-pharmacologic interventions have gained notice in CLBP management, offering potential benefits while reducing the risks due to pharmacologic treatments. These interventions include traditional (cognitive-behavioral therapy (CBT), physical activity, education) and non-traditional therapies (acupuncture, energetic therapies, practices based on body manipulation, body-mind interventions) [5–8].

An important aspect of CLBP research is the identification and evaluation of biomarkers, which are measurable biological indicators reflecting physiologic and/or molecular changes related to diseases [9, 10]. The biomarker assessment furnishes researchers with a mechanism to elucidate the intricate pathogenesis of CLBP and appraise the efficacy of interventions [11]. These biomarkers, particular inflammatory ones such as tumor necrosis factor-alpha (TNF-α), have become central to understanding CLBP’s pathogenesis and evaluating treatment efficacy [12, 13]. For those common inflammatory biomarkers, TNF-α, Interleukin (IL)-1, IL-1β, IL-2, IL-6, IL-8, and Interferon-gamma (IFNγ) refer to the pro-inflammatory cytokines, which are primarily synthesized by activated macrophages and play a pivotal role in the enhancement of inflammatory responses [14]. Besides, IL-4 and IL-10 refer to the anti-inflammatory cytokines, which encompass a series of immunoregulatory responsible for modulating the pro-inflammatory cytokine cascade [14]. Cytokines are small secreted proteins released by cells that elicit specific effects on intercellular interactions and communication. The interplay of pro-inflammatory cytokine signaling among immune, glial, and neural cells plays a fundamental role in the genesis of pathological pain [15].

Classic pro-inflammatory cytokines (TNF-α, IL-1β, IL-6, IL-8, and C-reactive protein (CRP)) have been reported to be increased in CLBP patients in some studies [10, 16]. And on the aspect of pain perception, some of these inflammatory biomarkers (TNF-α, IL-6, IL-1β, IL-2, and IFNγ) have shown correlations with pain intensity in chronic pain (neuropathic pain, arthrosis, back pain, and chronic musculoskeletal pain) [13, 17]. Interferon-γ-induced protein 10-γ-induced protein 10 (IP-10), Chemokine ligand (CCL) 2, CCL3, and CCL4 belong to chemokines, also known as chemotactic cytokines, which are a family of small proteins that play a crucial role in immune response and inflammation [18], and are related to pain transmission [19]. Research found an increased CCL2 and CCL3 production in patients with chronic and recurrent neck pain [20]. Moreover, the increased levels of CCL2, CCL4 and higher trend of CCL3 have also been found in CLBP patients [21], suggesting that chemokines may play a role in the pain process.

Notably, the non-pharmacologic treatments for CLBP, such as exercise, provides modulation of inflammatory biomarkers [22], frequent exercise not only decreases pro-inflammatory cytokines like TNF-α but also increases anti-inflammatory cytokines such as IL-10 [23]. Resistance exercises might modify the intervertebral disc metabolism, improving exchange, in this way favoring the repair of the lumbar discs [24]. As a parallel example, a systematic review of Fibromyalgia suggests that exercise acts as anti-inflammatory through pro-inflammatory cytokines [25].

Considering the established interplay between CLBP and inflammatory biomarkers, and the modulatory effects of non-pharmacologic interventions. This study aims to focus on the changes in inflammatory biomarkers following non-pharmacologic interventions in the context of CLBP patients, enhancing the scientific understanding of the complex interactions between non-pharmacologic interventions and inflammatory biomarker changes in CLBP, and hopefully contributing to the precision of therapeutic strategies.

## Methods

This review follows the PRISMA 2009 checklist [26] and the PICOT schema.

### Eligibility criteria

In terms of eligibility criteria, a study must have fulfilled the following inclusion criteria to be included in this review.

#### Population

Inclusion criteria: adult patients (age > 18), of both sexes, with (exclusively) chronic low back pain persists or recurs for more than 3 months. This fits to the new classification “chronic primary musculoskeletal pain” of International Classification of Diseases (ICD) 11 MG30 or to the old classification ICD 10 M54 (unspecific chronic low back pain) [27]. Excluded were other pain conditions associated with significant trauma or surgery such as chronic cancer-related pain, chronic postsurgical or posttraumatic pain, chronic neuropathic pain (radicular), or chronic secondary visceral pain, [28] as well as any other chronic secondary musculoskeletal pain [29] with specific diagnoses such as spinal stenosis, degenerative disc disease, disc herniation [30], spondylarthrosis (autoimmune), pregnancy or postpartum low back pain will be excluded.

#### Intervention

Inclusion of non-invasive non-pharmacologic interventions for chronic low back pain, defined as procedures to reduce symptoms and promote well-being [31], with therapy duration of more than one day at least such as exercise, manual & manipulative treatment, non-invasive acupuncture, massage, mind–body interventions (yoga, tai chi, mindfulness-based stress reduction, etc.), psychological and rehabilitation therapies [32]. Exclusion of dietary interventions and invasive interventions such as Pulsed Radiofrequency and Spinal cord stimulation implantation.

Considering the reality that non-pharmacologic interventions are sometimes used in conjunction with pharmacologic treatments in clinical sitting, studies including pharmacologic intervention factors will be handled in the following manner:

- If non-pharmacologic interventions are used alongside pharmacologic interventions, the impact of non-pharmacologic interventions must be the primary research objective of the included studies, and this will be clearly stated during the analysis phase, and the potential impact on the results will be discussed.
- If pharmacologic intervention is the main intervention as methods and non-pharmacologic intervention is only used as an adjunctive treatment, such studies will be excluded.
- In interpreting the results, special attention will be paid to the potential impact of pharmacologic interventions on non-pharmacologic interventions.

#### Comparison

No Intervention, other non-pharmacologic intervention, pharmacologic intervention or no comparator is allowed.

#### Outcome

Inclusion of studies reporting at least one inflammatory response biomarker measured in blood. In this review, only blood-based biomarkers well-known to be directly involved in inflammatory processes in the included studies were taken into consideration [30]. And these biomarkers were divided into pro-inflammatory cytokines (TNF-α, IL-1, IL-1β, IL-2, IL-6, IL-8, IFNγ), anti-inflammatory cytokines (IL-4, IL-10), acute-phase protein (CRP), and chemokine (IP-10, CCL2, CCL3, CCL4).

#### Type of study design

Experimental, Intervention (longitudinal) studies (Trials):

- Randomized controlled trials (RCTs).
- Non-randomized studies of interventions (N-RCTs).
  - Controlled (comparative): Cohort, case-control, controlled before-and-after, interrupted time series, controlled quasi-randomized.
  - No controlled (No comparative): Before-and-after.

The classification of study designs follow the National Institute for Health and Care Excellence public health guidelines [33]. Furthermore, the exclusion of reviews, protocols without results, or editorials was intended.

### Information sources

A systematic review from January 1st, 2002 to October 5th, 2022 in the following electronic databases was conducted: PubMed, Medline (platform Web of Science), and the Cochrane Library (platform Wiley Online Library). The search terms were “inflammatory biomarkers”, “back pain”, and “interventional studies”. We used Medical Subject Headings or synonyms around the key search words for the adaptation to each database (see Appendix 1). The references list of the identified original articles and reviews were searched manually for including the additional studies. The systematic review was conducted on October 5th, 2022, and the electronic search was done not exceed 7 days. Studies published in English, German, and Spanish published from January 1st, 2002 to October 5th, 2022 were included. Considering that there is an overlap of ICD-10 and ICD-11 in this time period, both disease classifications were under our consideration.

### Study selection process

Two reviewers (LPV and YH) screened each record, in the first phase reading the title and abstract, and afterward performing a full-text assessment. The methods of data extraction from reports were conducted using the same data collection adapted form based on Cochrane authors resources [34] for every study, containing the following items:

- Information about the study (author(s), year of publication, title, citation).
- Demographics (age, sex, diseases/conditions, baseline pain).
- Methodology (study design, participant recruitment (setting) / selection / allocation, level of evidence, information for the risk of bias study quality assessment).
- Intervention & Comparator (duration, time frame, setting).
- Outcomes description (method of analysis, time points measured).
- Results (sample sizes, pre-test data, post-test data, follow-up data, statistical tests used). Main summary measures are differences in means with their respective standard deviation.

Disagreements between authors were resolved via discussion, and consensus was reached without the need for arbitration by a third reviewer.

### Risk of bias in individual studies

The risk of bias assessment was conducted using two different tools depending on the study design; RoB 2 from Cochrane [35] or an adaptation of the Downs and Black checklist [36].

- Randomized controlled trials (RCTs).

For the RCTs, the risk-of-bias RoB 2 Tool from the Cochrane collaboration was employed [35].

- Non-randomized studies of interventions (N-RCTs).
- Considering that the ROBINS-I Tool Cochrane collaboration recommended for N-RCTs [37] do not include the no controlled (Before-and-after) studies, and modified adapted version of the Downs and Black checklist has been employed in the current study.
- The Downs and Black checklist aims to evaluate the methodological quality of both randomized and non-randomized comparative studies. This checklist comprises 27 items, distributed over reporting (questions 1–10), external validity (questions 11–13), internal validity - bias (questions 14–20), internal validity - confounding (questions 21–26), and power (question 27) [36]. Some reviews in the field of sports science employed modified versions of the Downs and Black checklist. In our review, a ‘Not applicable’ (N/A) was added for the non-controlled (Non-comparative) studies as a fourth option for items 5, 14, 15, 17, 19, 21, 22, 23, 24 and 25, which rating were excluded from the final assessment [38].
- Further, because of ambiguity in the ‘power’ item [39], and considering that most of the included studies referred to a subsample or a pilot study, the power calculation would not be contemplated in the quality assessment and would be reported as “Not applicable” for all of the studies.
- The original Downs and Black checklist does not have a pre-specified cut-off for acceptable studies [39]. So quality cut-off points were decided on retrospectively and studies were ranked to be of low (< 50%), moderate (51–75%), or good (76–100%) methodological quality [38]. The non-controlled studies, before-and-after studies were excluded from the analysis on the level of evidence [38] when having ‘Not applicable’ in most of the items from internal validity - bias.

The risk of bias in individual studies was used qualitatively for the data synthesis and discussion.

## Results

### Study selection

**Fig. 1:**
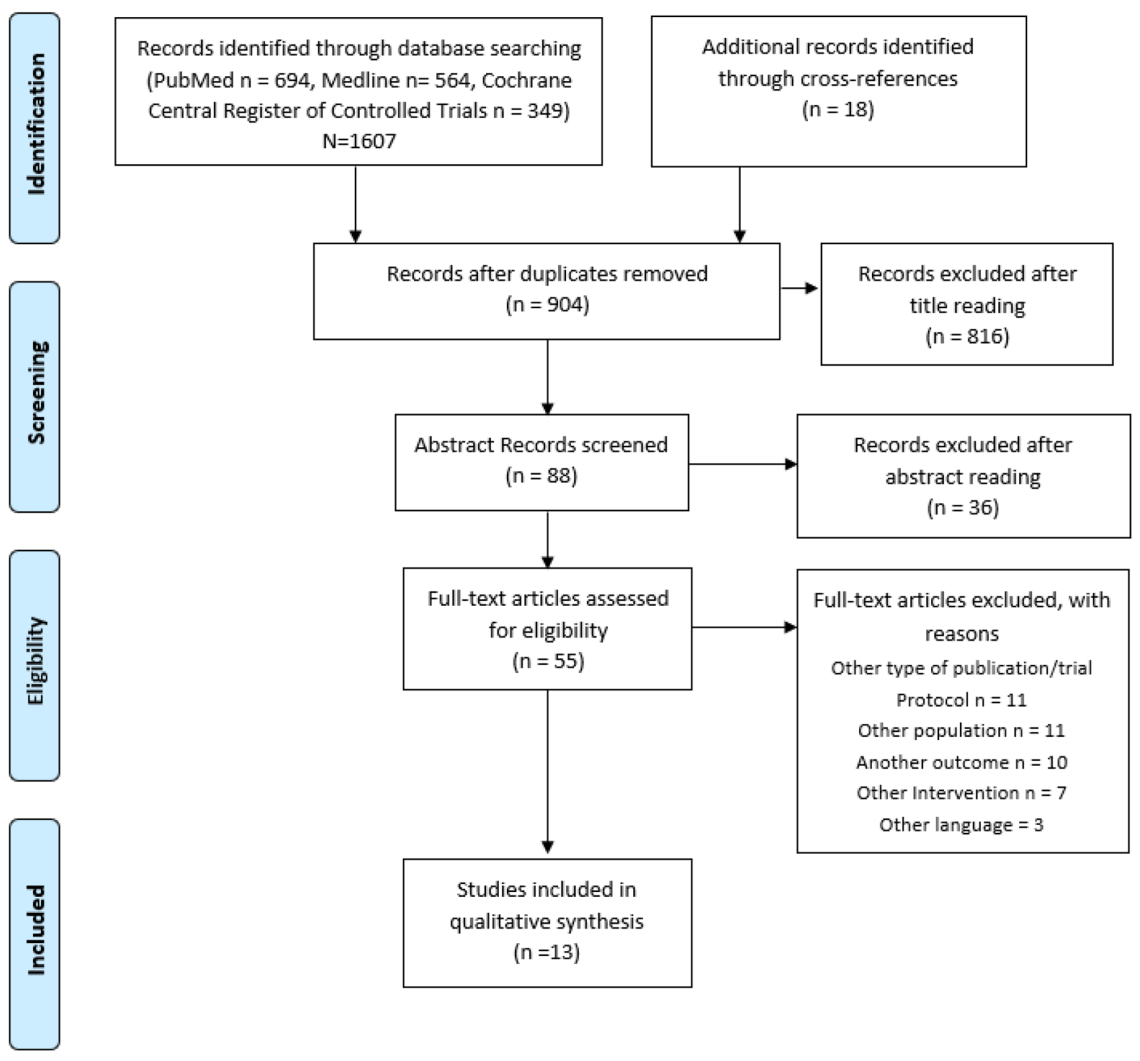
Flow chart studies included

Initially 1607 records were identified through the three databases, from which 55 were assessed via full text for eligibility and five were included in the systematic review (see Fig. 1).

### Study characteristics

A total of 13 studies were included in the systematic review, eight RCTs, one quasi-randomized trial, and four before-after studies. The sample size of the studies was small (*n* = 532 of the total participants in all included studies), eight studies analyzed up to *n* = 30 participants, four RCTs were *n* = 50 to 70 participants and only one RCT had a bigger sample size with *n* = 103.

The interventions studied consisted of manual and manipulative treatment (four studies, one osteopathic manual treatment (OMT) based, and three spinal manipulative therapy (SMT) based), exercise (two studies), yoga (two studies), and acupressure (two studies). Further, one neuro-emotional technique (NET), one mindfulness-based, and one balneotherapy study was integrated. Various blood-based common inflammatory biomarkers were assessed; Interleukins IL-1, IL-2, IL-4, IL-6, IL-8, IL-10, TNF-α, IFNγ, CRP and CCL2, CCL3, and CCL4. See Table 1. Characteristics of the Studies included. Four studies reported some changes in the inflammatory biomarkers compared to the control group. Decreased TNF-α after OMT, NET, and yoga. Decreased IL-1, IL-6, IL-10, and CRP after NET, and increased IL-4 after acupressure. Another five studies found changes in inflammatory biomarkers through pre- and post-intervention comparisons. Increased IL-10 after balneotherapy; decreased TNF-α, IL-1β, IL-8, IFNγ, IP-10 after exercise; decreased IL-6 after exercise and SMT; decreased CRP and chemokine ligand 3 after SMT.

**Table 1:** Characteristics of the Studies included

Authors	Study design	Population	Sample	Intervention (I) / Comparator (C)	The change of Inflammatory biomarkers	Pharmacologic interventions
**Study design: RCTs**						
Bablis et al. 2022 [45]	RCT	Non-specific CLBP	N_R_ = 173 (I:87; C:86) N_T0_ = 112 (I:58; C:54) N_T1−T3_ = 103 (I:54; C:49) T1: 1 month after T0 (TA) T2: 3 months after T0 T3: 6 months after T0	**I** = Neuro-emotional technique **C** = Sham neuro-emotional technique Duration: 2x weekly for 4 weeks.	TNF-α ↓ (significant decrease in **I** but not in **C**) IL-1 ↓ (significant decrease in **I** but not in **C**) IL-6 ↓ (significant decrease in **I** but not in **C**) IL-10 ↓ (significant decrease in **I** but not in **C**) CRP ↓ (significant decrease in **I** but not in **C**) (Serum)	no
Licciardone et al. 2012 [49]	RCT	Non-specific CLBP	Sub-study nested N_T0_ = 70 (I:38; C:32) N_T1_ = 55 (I:28; C:27) T1: 12 weeks after T0 (TA)	**I** = Osteopathic manual treatment **C** = Sham osteopathic manual treatment Duration: 6 × (15 min/session) for 8 weeks.	TNF-α ↓ (significant decrease in **I** than in **C**) IL-1β (no significant change between **I** and **C**) IL-6 (no significant change between **I** and **C**) IL-8 (no significant change between **I** and **C**) IL-10 (no significant change between **I** and **C**) (Serum)	21/38 of **I** used nonprescription drugs for LBP in previous 4 weeks 3/38 of **I** used prescription drugs for LBP in previous 4 weeks 18/32 of **C** used nonprescription drugs for LBP in previous 4 weeks 8/32 of **C** used prescription drugs for LBP in previous 4 weeks No statistically difference of drugs use between **I** and **C**
Lin et al. 2015 [40]	RCT	CLBP	Sub-study nested N_T0−T1_ = 61 (I:30; C:31) T1: 4 weeks after T0 (TA)	**I** = Auricular point acupressure **C** = Sham auricular point acupressure Duration: 1x weekly for 4 weeks.	TNF-α (no significant change between **I** and **C**) IL-1β (no significant change between **I** and **C**) IL-2 (no significant change between **I** and **C**) IL-4 ↑ (significant increase in **I** than in **C**) IL-6 (no significant change between **I** and **C**) IL-10 (no significant change between **I** and **C**) (Serum)	13/30 (43%) of **I** and 14/31 (45%) of **C** have the current pain medication use No statistically difference of current pain medication use between **I** and **C** (*p* = 0.89)
Nambi et al. 2020 [52]	RCT	Non-specific CLBP	N_R−T0_ = 60 (I_1_:20; I_2_:20; C:20) N_T1_ = 59 (I_1_:19; I_2_:20; C:20) T1: 4 weeks after T0 (TA)	**I**_**1**_ = Isokinetic training **I**_**2**_ = Core stabilization training **C** = Conventional balance training for core muscles Duration: 5x weekly for 4 weeks.	Results not reliable, inconsistency in all the article	No
Poojari et al. 2022 [41]	RCT	CLBP	N_R−T0_ = 35 (I:17; C:18) N_T1_ = 31 (I:14; C:17) N_T2_ = 29 (I:14; C:15) T1: 1 month after T0 T2: 3 months after T0 (TA)	**I** = Integrated Approach of Yoga Therapy + Usual care. - Center-based: 6x weekly (60 min/session) for 2 weeks. - Home-based: suggested daily (45 min/session) until 3 months. **C** = Usual care (Education on disease, self-care, physically activity, lifestyle and a back-care booklet) Duration: 3 months	TNF-α (no significant change between **I** and **C**) (Serum)	6/14 (42.9%) of **I** have monotherapy for CLBP 8/14 (57.1%) of **I** have dual therapy for CLBP 9/15 (60%) of **C** have monotherapy for CLBP 6/15 (40%) of **C** have dual therapy for CLBP No statistically difference of drug therapy between **I** and **C** (*p* = 0.356)
Yeh et al. 2014 [42]	RCT	CLBP	N_T0−T1−T2_ = 19 (I:10; C:9) T1: 4 weeks after T0 (TA) T2: 1 month after T1	**I** = Auricular point acupressure **C** = Sham auricular point acupressure Duration: 4 weeks	TNF-α (no significant change between **I** and **C**) IL-1β (no significant change between **I** and **C**) IL-2 (no significant change between **I** and **C**) IL-4 (no significant change between **I** and **C**) IL-6 (no significant change between **I** and **C**) IL-10 (no significant change between **I** and **C**) (Serum)	2/10 (20%) of **I** use pain medication at baseline 4/9 (44%) of **C** use pain medication at baseline
Yücesoy et al. 2021 [44]	RCT	Non-specific CLBP	N_R−T0_ = 74 (I:37; C:37) N_T1_ = 66 (I:33; C:33) T1: 2 weeks after T0 (TA)	**I** = Balneotherapy (5x weekly) + Home exercise program (daily) **C** = Home exercise program (daily) Duration: 2 weeks	IL-6 (no significant change after **I**) IL-10 ↑ (significant increase after **I**) (Serum)	The usage of analgesics: 0.94 (number/day) of **I**, 1.94 of **C** used NSAID in T1. No statistically difference of NSAID usage between **I** and **C** (*p* = 0.458) 0.33 of **I**, 0.46 of **C** used Paracetamol in T1. No statistically difference of Paracetamol usage between **I** and **C** (*p* = 0.76)
Zgierska et al. 2016 [43]	RCT	CLBP	N_R−T0_ = 35 (I:21; C:14) N_T1_ = 32 (I:18; C:14) N_T2_ = 28 (I:15; C:13) T1: 8 weeks after T0 T2: 26 weeks after T0 (TA)	**I** = Meditation combined with cognitive behavioral therapies + Usual care. - Center-based: 2 h weekly for 8 weeks - Home-based: suggested mindfulness meditation 6 days/week (at least 30 min) until 26 weeks. **C** = Usual care (pharmacotherapy, monitoring, referral to specialty care) Duration: 6 days/week (at least 30 min) for 26 weeks.	TNF-α (no significant change between **I** and **C**) IL-1β (no significant change between **I** and **C**) IL-6 (no significant change between **I** and **C**) IFNγ (no significant change between **I** and **C**) CRP (no significant change between **I** and **C**) (Serum)	Mean (Standard deviation) of opioid dose: 166.9 (153.7) in **I**, 120.3 (76.9) in **C** No statistically difference of opioid dose between **I** and **C** (*p* = 0.654)
**Study design: Non-Randomized studies of interventions**						
Cho et al. 2015 [53]	Controlled - quasi-randomized trial	Non-specific CLBP	N_E_ = 43 (I:23; C:20) N_T1_ = 24 (I:14; C:11) T1: 12 weeks after T0 (TA) N_IT_= 43 (I:23; C:20)	**I** = Hatha yoga [26]: 3x weekly **C** = No treatment Duration: 12 weeks.	TNF-α ↓ (significant decrease in **I** than in **C**) CRP (no significant change between **I** and **C**) (Serum)	no
Cheng et al. 2015 [54]	No controlled - Before-and-after study	Non-specific LBP	N_T0−T1_ = 30 T1: 4 weeks after T0 (TA)	**I** = Exercise training: 3x weekly Duration: 4 weeks.	TNF-α ↓ (significant decrease after **I**) IL-1β ↓ (significant decrease after **I**) IL-6 ↓ (significant decrease after **I**) IL-8 ↓ (significant decrease after **I**) IFNγ ↓ (significant decrease after **I**) IP-10 ↓ (significant decrease after **I**) (Plasma)	no
Roy et al. 2010 [55]	No controlled - Before-and-after study	CLBP	N_T0_ = 11 N_T1_ = 10 T1: 2 weeks after T0 (TA)	**I** = Chiropractic spinal manipulative therapy Duration: 9 sessions for 2 weeks. (Negative control group: With no LBP or treatment N_T0−T1_ = 10 was used as a reference)	IL-6 ↓ (significant decrease after **I**) (Plasma) CRP ↓ (significant decrease after **I**) (Serum)	no
Teodorczyk-Injeyan et al. 2018 [21]	No controlled - Before-and-after study	Non-specific CLBP	N_T0−T1_ = 23 T1: 2 weeks after T0 (TA)	**I** = Spinal manipulative therapy Duration: 6 sessions for 2 weeks. (Negative control group: With no LBP or treatment N_T0−T1_ = 21 was used as a reference)	CCL2 (no significant change after **I**) CCL3 ↓ (significant decrease after **I**) CCL4 (no significant change after **I**) (blood)	no
Teodorczyk-Injeyan et al. 2021 [56]	No controlled - Before-and-after study	Non-specific CLBP	N_T0−T1_ = 25 T1: 2 weeks after T0 (TA)	**I** = Spinal manipulative therapy Duration: 6 sessions for 2 weeks. (Negative control group: With no LBP or treatment N_T0−T1_ = 24 was used as a reference)	TNF-α (no significant change after **I**) IL-1β (no significant change after **I**) IL-2 (no significant change after **I**) IL-6 ↓ (significant decrease after **I**) IL-10 (no significant change after **I**) IFNɣ (no significant change after **I**) (blood)	no

“↑” means increase; “↓” means decrease; N: Number of participants, total sample size of the population of interest.; N_R_ = Number of participants randomized; N_E_ = Number of participants enrolled; N_T0_ = Number of participants with baseline measurements; N_T1_ = Number of participants with T1 measurements; N_IT_ = Number of participants analyzed with Intention to treat analysis; NSAID: non-steroidal anti-inflammatory drug; TA: Timepoint direct after Intervention; RCT: Randomized controlled trial; CLBP: Chronic Low Back Pain; LBP = Low back pain; IL-1β = Interleukin 1β; IL-2 = Interleukin 2; IL-4 = Interleukin 4; IL-6 = Interleukin 6; IL-8 = Interleukin 8; IL-10 = Interleukin 10; IP-10 = interferon-γ-induced protein 10-γ-induced protein 10; TNF-α = Tumor necrosis factor alpha; CRP = C-reactive protein; IFNγ = Interferon-gamma; CCL2 = chemokine ligand 2; CCL3 = chemokine ligand 3; CCL4 = chemokine ligand 4

**Fig. 2:**
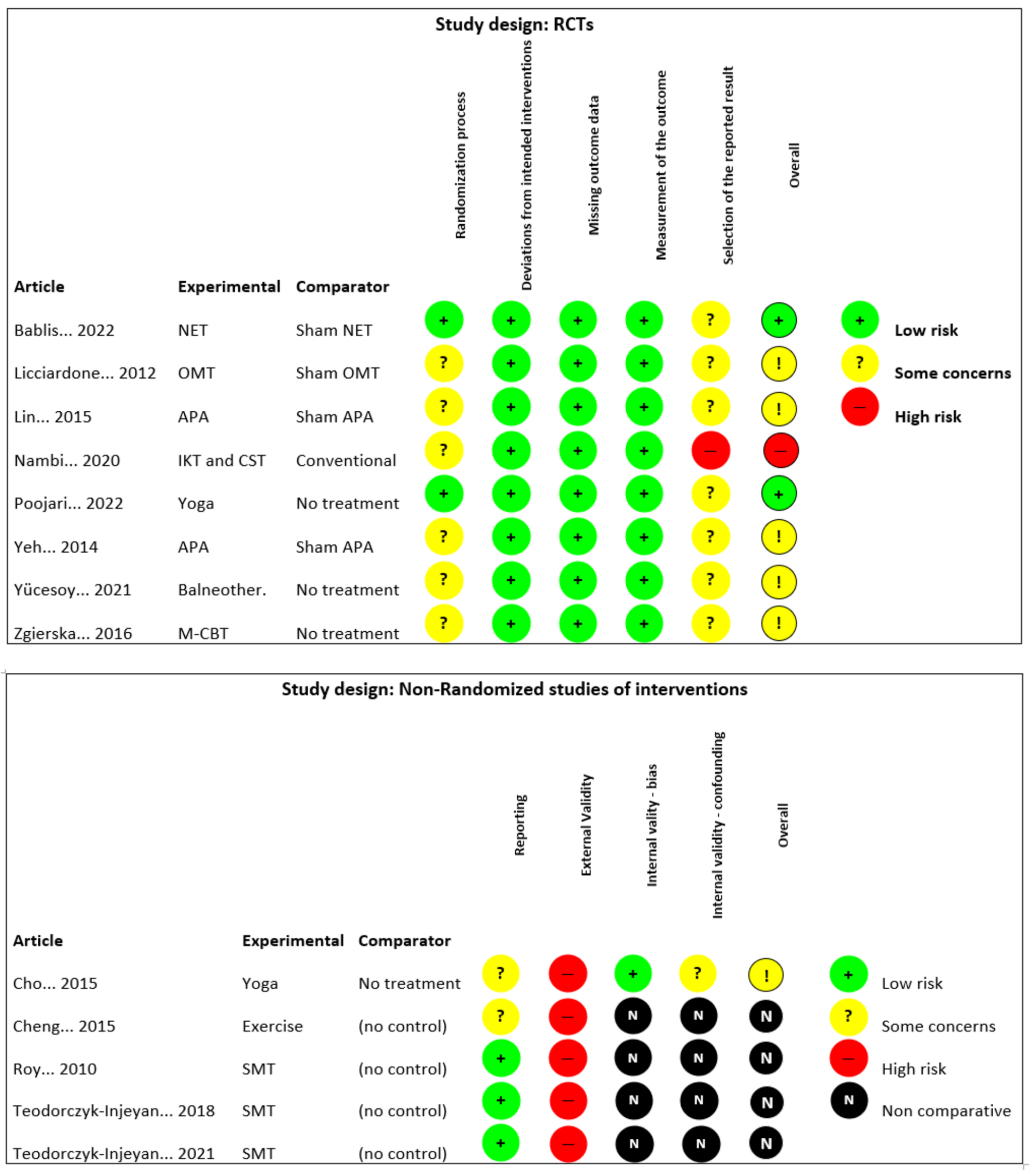
Quality assessment of the studies included. NET: neuro-emotional technique; OMT: Osteopathic manual treatment; APA: Auricular Point Acupressure; IKT: Isokinetic training; CST: Core stabilization training; M-CBT: Meditation-cognitive behavioral therapy; SMT: spinal manipulative therapy

Two RCTs were ranked as having good methodological quality, five studies had some concerns in the assessment, one RCT had low quality, and no quality label was attached to the four before-after studies due to the lack of qualification in the internal validity (bias) section (See Fig. 2. Quality assessment).

### Handling of pharmacologic interventions

From all 13 included articles, 6 RCT articles reported non-pharmacologic interventions are used alongside pharmacologic interventions, and 5 of these expressly stated that the dose or number of users of the pharmacologic intervention was not statistically different between the intervention and comparison groups. 2 RCTs, 1 quasi-randomized trial, and 4 before-after studies exclude pharmacologic interventions.

### Results of individual studies

Concerning each inflammatory biomarkers changes or non-changes within the included studies, the following results were found:

### Pro-inflammatory cytokines

- **TNF-**α

From the 10 articles considering TNF-α changes, 7 compare the groups time changes (time*group comparison), so from these 7 studies, three studies found a statistically significant greater reduction in the intervention group as in the control group: these studies had NET, OMT, and yoga as intervention. Moreover, considering only TNF-α changes after intervention without regarding the control group (2 studies) one extra study (exercise) indicate a reduction of this biomarkers after the intervention. In addition to these significant changes, four RCTs [40–43] did not found a statistically significant change difference in TNF-α between intervention and control. Two RCTs [40, 42] analyzed acupressure, another one yoga in CLBP patients not responding to conservative management [41], while the last one, studied Meditation-cognitive behavioral therapy (M-CBT) in a CLBP subpopulation with high baseline pain levels (morphine prescription) [43].

- **IL-1**

Of all the literature finally included, only one tested for IL-1, it found a statistically significant greater reduction in the intervention group as in the control group with the NET intervention.

- **IL-1β**

From 6 studies, 4 reported time*group comparisons, and none found a statistically significant greater reduction in the intervention group as in the control group. Moreover, considering only IL-1β changes after intervention without regarding the control group (2 studies), 1 study (exercise) found a significant reduction IL-1β level after intervention.

- **IL-2**

From 4 studies, none found a statistically significant greater reduction in the intervention group as in the control group or reductions after the intervention.

- **IL-6**

From 10 studies, 5 reported time*group comparisons, and only 1 found a statistically significant greater reduction in the intervention group as in the control group with the NET intervention. Moreover, considering only IL-6 changes after intervention without regarding the control group, 3 extra studies (exercise and SMT) indicated a reduction of this biomarker after the intervention.

- **IL-8**

The one study reporting time*group comparisons did not found a statistically significant greater reduction in the intervention group as in the control group. Moreover, a reduction after the exercise intervention in 1 study was noted.

- **IFNγ**

From 4 studies, only one reported time*group comparisons, and did not found a statistically significant greater decrease in the intervention group as in the control group after the intervention. Moreover, only decreased IFNγ level was found after intervention (exercise) without regarding the control group.

### Anti-inflammatory cytokines

- **IL-4**

From 3 studies, one found a statistically significant greater increase in the intervention group than control group after the intervention. The intervention was Auricular Point Acupressure (APA).

- **IL-10**

From 6 studies, 4 reported time*group comparisons, none found a statistically significant greater increase in the intervention group than control group after the intervention. Moreover, considering only IL-10 increases after intervention without regarding the control group, 1 extra study (Balneological treatment) indicated an increase of this biomarker after the intervention.

### Acute-phase protein

- **CRP**

From 5 studies, three reported time*group comparisons and one found a statistically significant greater reduction in the intervention group than control group with the NET intervention. Moreover, reduction after the SMT intervention in 1 study was noted.

### Chemokines

- **CCL2, CCL3, CCL4**

One study considering only chemokines changes after intervention without regarding the control group (evidence that only gives us only a tendency/hypothesis), report a reduction of CCL3 after the SMT intervention.

- **IP-10**

Only one study indicated a reduction of IP-10 after the exercise intervention without regarding the control group.

Moreover, in Sup. Table 1 is (see Appendix 2) possible to find the quantitative comparisons, within, between and time*group significant values with their conclusions.

## Discussion

This systematic review focus on the changes in inflammatory biomarkers following non-pharmacologic interventions in the context of CLBP patients, thus trying to understand the complex interactions between non-pharmacologic interventions and inflammatory biomarker changes in CLBP.

Our findings revealed a general trend towards reducing specific pro-inflammatory cytokines, such as TNF-α and IL-6, in response to interventions like NET, OMT, yoga, exercise, and SMT. Notably, TNF-α levels decreased consistently across multiple non-pharmacologic interventions. Similarly, CRP, which reflects ongoing inflammation and tissue damage, was reduced following NET and SMT interventions, suggesting a potential attenuation of the inflammatory response in CLBP patients. However, the findings for anti-inflammatory cytokines, such as IL-10, were more variable, with one study showing increased IL-10 levels after balneotherapy [44]. Another noticed a reduced IL-10 level [45], which may be related to the chronic course of CLBP, i.e., the expected increase of IL-10 may occur at an earlier stage of the low back pain episode, returning to normal levels over time specific to chronic disease. This difference highlights the complexity of the inflammatory response of CLBP and the varying effectiveness of non-pharmacologic interventions.

In terms of chemokines, our review found evidence that IP-10 and CCL3 are reduced following specific interventions (exercise and SMT). Consider chemokines induce inflammation, and the chemokine system governs not only inflammation within the immune system but also orchestrates neuroinflammation in both the peripheral and central nervous systems, contributing to the initiation and perpetuation of diverse chronic pain conditions [46]. It seems non-pharmacologic treatments can influence inflammation or even manage pain by lowering chemokine levels. But there are still some concerns that need to be addressed. Firstly, chemokine levels did not return to those observed in symptom-free patients, implying a short course (2 weeks) of SMT intervention may not be sufficient to resolve the tissue irritation associated with CLBP [21], which suggested an appropriate intervention period remains to be determined. Secondly, as only two articles have addressed the effect of non-pharmacologic interventions on chemokine levels in CLBP, more research is still needed regarding the specific role chemokines play during the intervention process in CLBP.

Previous systematic reviews have highlighted the role of non-pharmacologic interventions in managing inflammation in chronic diseases [47, 48], which shows similar outcomes explored by our review. Moreover, they highlighted the pain improvement of various non-pharmacologic interventions via modulating the inflammatory cytokine levels [48], providing a broader context for our findings and expecting the potential clinical relevance of these interventions. On this basis, our review adds specific knowledge by examining a range of interventions and their impact on inflammatory biomarkers in CLBP, which provides more information to understand how these interventions alleviate inflammation in this situation.

Regarding the reason for our findings, many factors need consideration. The different interventions and biomarkers led to a noticeable heterogeneity, partly explaining the differing degrees of impact on inflammatory biomarkers observed. For example, some interventions, such as NET and exercise, consistently reduced multiple pro-inflammatory cytokines and chemokines, but others had more variable effects. The heterogeneity also reflects different study quality across the included studies, with a mix of RCTs and N-RCTs, varying intervention durations, and differing sample sizes. This diversity could contribute to the observed differences in inflammatory biomarkers’ responses to various interventions and reflect the complex nature of CLBP itself.

In particular, we noted the non-pharmacologic interventions are used alongside pharmacologic interventions in 6 of the included RCTs. Notably, 5 of these studies specified no statistical difference in the dose or number of users of the pharmacologic intervention between the intervention and comparison groups. This finding is critical in reducing the potential bias of pharmacologic interventions when assessing the effects of non-pharmacologic interventions. Since pharmacologic interventions were consistent across groups, we can be more confident that the observed effects of non-pharmacologic interventions (e.g., OMT or Balneotherapy) are more likely to be independent of pharmacologic treatment.

However, even if pharmacologic interventions are consistent across groups, their presence may still have some impact on the effects of non-pharmacologic interventions. For example, medications may have provided some degree of pain relief, which may have affected patients’ motivation or ability to participate in non-pharmacologic treatments such as yoga. Therefore, it remains essential to consider potential interactions between pharmacologic and non-pharmacologic interventions when interpreting the results of these studies.

From a research perspective, this review revealed the ability of non-pharmacologic interventions to regulate inflammation and emphasized the potential of these interventions as therapies for treating CLBP. Admittedly, this review does not consider assessing clinical outcomes after non-pharmacologic interventions for CLBP, so the guidance for the clinic is limited. And the variability in study outcomes calls for cautious interpretation and more robust research to establish clear clinical guidelines.

In terms of limitations, it is to resume, that the number of included studies is limited, only 13, as well as their sample size. Apart from the study analyzing 103 total participants [45], the studies potentially had low detection power (high type II error); no calculation of power for the specific hypothesis regarding the inflammatory biomarkers is reported in any of the included studies. Further, due to the exploratory nature of some of the studies, not adjusting for multiple comparisons (analysis involving more than 1 biomarker) is reported (possible type I error), like in the OMT study [49]. The differences in inflammatory biomarkers we collected are statistically significant, but the evidence is still limited in number and methodological quality, which brings challenges in drawing definitive conclusions. Nonetheless, retention and recruitment during clinical trials often consume a considerable workload [50], especially when blood samples are required to be collected more than once. And the interpretation of the Non-Randomized studies of interventions (five studies), mainly because of the lack of a comparator (four before-and-after studies), has to be very cautious. A comparator group allows to differentiate the effects of the intervention from other factors like patient expectations, natural disease progression or another treatment [51].

Nevertheless, the review gives a broad view of this specific topic, and the systematic search has a high sensitivity; it was conducted in three state-of-the-art databases with an initial retrieval of more than 1600 records. The risk of bias assessment followed the up-to-date recommendations from the Cochrane’s reviews, and specific detailed quantitative data of each biomarker are displayed and analyzed in the results and discussion.

## Conclusion

The findings of this systematic review demonstrate a trend that different non-pharmacologic interventions targeting CLBP modulate inflammatory biomarkers. Most of the effects of these interventions on inflammatory mediators are reflected in decreased pro-inflammatory markers and increased anti-inflammatory markers. However, challenges exist due to the heterogeneity of patient’s populations and biomarkers measure methods, as well as considering the limited number of high-quality studies evaluating similar intervention methods and biomarkers, which limits the conclusion of this review.

### Electronic supplementary material

Below is the link to the electronic supplementary material.

Supplementary Material 1

Supplementary Material 2

## Data Availability

All data generated or analysed during this study are included in this published article and its supplementary information files.

## Abbreviations

RCT: Randomized controlled trial
N-RCT: Non-randomized studies of intervention
CLBP: Chronic low Back Pain
N: Number of participants, total sample size of the population of interest
NSAID: non-steroidal anti-inflammatory drug
NET: neuro-emotional technique
OMT: Osteopathic manual treatment
SMT: spinal manipulative therapy
ICD: International Classification of Diseases
IL: Interleukin
IP-10: interferon-γ-induced protein 10-γ-induced protein 10
TNF-α: Tumor necrosis factor alpha
CBT: Cognitive behavioral therapy
CRP: C-reactive protein
IFNγ: Interferon-gamma
CCL: Chemokine ligand
IKT: Isokinetic training
M-CBT: Meditation-cognitive behavioral therapy
CST: Core stabilization training
LBP: Low back pain
