# Supplementary material for: The changes of blood-based inflammatory biomarkers after non-pharmacologic interventions for chronic low back pain: a systematic review
Source: BMC Musculoskelet Disord. 2024 Mar 8;25:209. doi: 10.1186/s12891-024-07289-1 (PMC10921684; doi:10.1186/s12891-024-07289-1)
Supplement: Supplementary file 1 — Supplementary Material 1 [file 12891_2024_7289_MOESM1_ESM.docx]

**Appendix 1. Specific search string, search databases**

**Databases**

- **PubMed (05.10.2022)**

(("Immunologic Factors"[Mesh] OR "Immunologic Tests"[Mesh] OR "immunologic" OR "inflammat*") **AND** ("Biomarkers"[Mesh] OR "Biomarkers, Pharmacological"[Mesh] OR "marker*" OR "factor*" OR "blood" OR "plasma" OR "serum")) **AND** ("Back Pain"[Mesh] OR "Low Back Pain"[Mesh] OR "back pain") **NOT** ("review"[Publication Type] OR "review literature as topic"[MeSH Terms] OR "review") AND ("trial*" OR "intervention*" OR "programm*" OR "effect*")

Records: After filter “Last 20 years” - **694**

- **Medline (platform Web of Science) (05.10.2022)**

**("immunologic" OR "inflammat*")**(Topic)**AND ("marker*" OR "factor*" OR "blood" OR "plasma" OR "serum")** (Topic)**AND ("back pain")**(Topic)**AND ("trial*" OR "intervention*" OR "programm*" OR "effect*")**(Topic)**NOT (Review OR Systematic Review OR Meta Analysis OR Bibliography OR Address OR Addresses OR Biography OR Comment OR Editorial OR Webcasts OR Webcast OR Retracted Publication OR Portraits OR Portrait OR News OR Letter OR Legislation OR Legal Cases OR Lecture OR Case Reports OR Video Audio Media) (Publication Type)**

Records: After filter “2002-2022” - **564**

- **Cochrane Central Register of Controlled Trial (05.10.2022)**

("immunologic" OR "inflammatory" OR "inflammation") **AND** ("marker" OR "markers" OR "factor" OR "factors" OR "blood" OR "plasma" OR "serum") **AND** ("back pain")

Records: After filter “Trials” and **“**last 20 years” - **349**
