# Supplementary material for: The changes of blood-based inflammatory biomarkers after non-pharmacologic interventions for chronic low back pain: a systematic review
Source: BMC Musculoskelet Disord. 2024 Mar 8;25:209. doi: 10.1186/s12891-024-07289-1 (PMC10921684; doi:10.1186/s12891-024-07289-1)
Supplement: Supplementary file 2 — Supplementary Material 2 [file 12891_2024_7289_MOESM2_ESM.docx]

| **Sup.Table 1. Quantitative results included studies, pro biomarker** | | | | | |
| --- | --- | --- | --- | --- | --- |
|  | | **Results** | | | |
| **Biomarker** | **Article** | **Quantitative comparisons** | | | **Qualitative conclusions** |
|  |  | **within** (T_A_ – T_0_) | **between, at T**_A_ (C – I) | **time*group** *(∆ I - ∆ C)* |  |
| **TNF-α (pg/mL)**  *–*  *Proinflammatory:*  *reduction expected* | Bablis et al. 2022  (59) | *∆* *I :* -0.64 (clinically significant change)  *∆ C :* 0.22 (no significant change) | 0.87 [95%CI: 0.68, 1.06]; p ≤ 0.001 | (p ≤ 0.001) | - Clinically significant decrease in I but not in C |
|  | Licciardone et al. 2012 (60) | *∆* *I :* -1.06 [IQR: 2.11]  *∆ C :* -0.06 [IQR: 1.90] | NR | p = 0.03 | - Statistical significantly greater reduction in I than in C |
|  | Lin et al. 2015 (55) | *∆* *I :* 2.55 [SD: 24.39]  *∆ C :* -5.61 [SD: 22.96] | NR | 8.16 [SD: 23.71]; p = 0.20 | - No statistically significant change difference between I and C |
|  | Nambi et al. 2020 (61) | *∆* *I*_1_ *; ∆* *I*_2_ *; ∆* *C :* (increase) | NV | NR | - Results not reliable, inconsistency in all the article |
|  | Poojari et al. 2022 (56) | *∆* *I :* p = 0.004  *∆ C :* p = 0.002 | NR | p = 0.77 | - No statistically significant reduction difference between I and C |
|  | Yeh et al. 2014 (57) | *∆* *I :* 2.00 [IQR: 7.5]; p=0,69  *∆ C :* -5.00 [IQR: 11]; p=0,50 | p = 0.88 | NA | - No statistically significant change difference between I and C (or after treatments) |
|  | Zgierska et al. 2016 (58) | NR | 3.3 [95%CI: -2.5, 9.2] | p = 0.40 | - No statistically significant change difference between I and C |
|  | Cho et al. 2015 (63) | *∆* *I :* -0.1 [SD: 0.5]  (not significant difference)  *∆ C :* 1.4 [SD: 1.8] | NR | p < 0.01 | - Statistical significantly greater reduction in I than in C   (Intention to treat analysis) |
|  | Cheng et al. 2015 (64) | *∆* *I : (*reduction); p ≤ 0.05 | NA | NA | - Statistical reduction after I |
|  | Teodorczyk-Injeyan et al. 2021 (66) | *∆* *I :* (not significant difference) | NA | NA | - No statistically significant reduction after I |
|  | | | | | |
| **IL-1**  **(pg/mL)**  *–*  *Proinflammatory:*  *reduction expected* | Bablis et al. 2022 (59) | *∆* *I :* -1.07 (statistical significant change)  *∆ C :* 0.26 (no significant change) | 1.32 [95%CI: 0.84, 1.79]; p ≤ 0.001 | (p ≤ 0.001) | - Statistically significant decrease in I but not in C (no clinically significant) |
| **IL-1β (pg/mL)** | Licciardone et al. 2012 (60) | *∆* *I :* 0.00 [IQR: 0.00]  *∆ C :* 0.00 [IQR: 0.06] | NR | p > 0.05 | - No statistically significant change difference between I and C |
|  | Lin et al. 2015 (55) | *∆* *I :* -1.33 [SD: 5.00]  *∆ C :* -0.39 [SD: 2.09] | 0.94 [SD: 3.88] | p = 0.35 | - No statistically significant change difference between I and C |
|  | Yeh et al. 2014 (57) | *∆* *I :* 0.00 [IQR: 18]; p=0,55  *∆ C :* 0.00 [IQR: 1.5]; p=0,56 | p = 0.82 | NA | - No statistically significant change difference between I and C (or after treatments) |
|  | Zgierska et al. 2016 (58) | NR | -0.04 [95%CI: -0.6, 0.5] | p = 0.196 | - No statistically significant change difference between I and C |
|  | Cheng et al. 2015 (64) | *∆* *I :* (reduction); p ≤ 0.05 | NA | NA | - Statistical reduction after I |
|  | Teodorczyk-Injeyan et al. 2021 (66) | *∆* *I :* (not significant difference) | NA | NA | - No statistically significant reduction after I |
|  | | | | | |
| **IL-2**  *–*  *Proinflammatory:*  *reduction expected* | Lin et al. 2015 (55) | *∆* *I :* -1.24 [SD: 6.51]  *∆ C :* -0.39 [SD: 5.34] | 0.85 [SD: 5.98] | p = 0.58 | - No statistically significant change difference between I and C |
|  | Nambi et al. 2020 (61) | *∆* *I*_1_ *; ∆* *I*_2_ *; ∆* *C :* (increase) | NV | NR | - Results not reliable, inconsistency in all the article |
|  | Yeh et al. 2014 (57) | *∆* *I :* 0.00 [IQR: 5]; p=0,55  *∆ C :* 0.00 [IQR: 1.5]; p=0,59 | p = 1.00 | NA | - No statistically significant change difference between I and C (or after treatments) |
|  | Teodorczyk-Injeyan et al. 2021 (66) | *∆* *I :* (not significant difference) | NA | NA | - No statistically significant reduction after I |
|  | | | | | |
| **IL-4**  *–*  *Anti-inflammatory:*  *increase expected* | Lin et al. 2015 (55) | *∆* *I :* 0.40 [SD: 2.46]  *∆ C :* -0.93 [SD: 2.52] | 1.33 [SD: 2.49] | p = 0.05 | - Statistical significantly greater increase in I than in C (but exactly p = 0,05 and SD; between at T_A_, cross the zero) |
|  | Nambi et al. 2020 (61) | *∆* *I*_1_ *; ∆* *I*_2_ *; ∆* *C :* (increase) | NV | NR | - Results not reliable, inconsistency in all the article |
|  | Yeh et al. 2014 (57) | *∆* *I :* 0.00 [IQR: 1]; p=0,89  *∆ C :* 0.00 [IQR: 2]; p=0,66 | p = 0.94 | NA | - No statistically significant change difference between I and C (or after treatments) |
|  | | | | | |
| **IL-6 (pg/mL)**  *–*  *Proinflammatory: reduction expected* | Bablis et al. 2022 (59) | *∆* *I :* -2.05 (statistical significant change)  *∆ C :* 0.14 (no significant change) | 2.25 [95%CI: 1.32, 3.18]; p ≤ 0.001 | (p ≤ 0.001) | - Statistically significant decrease in I but not in C (no clinically significant) |
|  | Licciardone et al. 2012 (60) | *∆* *I :* 0.03 [IQR: 1.43]  *∆ C :* 0.03 [IQR: 0.47] | NR | p = 0.68 | - No statistically significant change difference between I and C |
|  | Lin et al. 2015 (55) | *∆* *I :* -2.18 [SD: 9.68]  *∆ C :* -1.28 [SD: 16.19] | NR | -0.90 [SD: 13.58]; p = 0.80 | - No statistically significant change difference between I and C |
|  | Nambi et al. 2020 (61) | *∆* *I*_1_ *; ∆* *I*_2_ *; ∆* *C :* (increase) | NV | NR | - Results not reliable, inconsistency in all the article |
|  | Yeh et al. 2014 (57) | *∆* *I :* -0.50 [IQR: 9]; p=0,95  *∆ C :* 0.00 [IQR: 7.5]; p=0,81 | p = 0.70 | NA | - No statistically significant change difference between I and C (or after treatments) |
|  | Yücesoy et al. 2021 (62) | *∆* *I :* (reduction but not significant)  *∆* C *:* (reduction but not significant) | NR | NR | - No statistically significant reduction after I |
|  | Zgierska et al. 2016 (58) | NR | 0.2 [95%CI: -1.3, 1.8] | p = 0.126 | - No statistically significant change difference between I and C |
|  | Cheng et al. 2015 (64) | *∆* *I : (*reduction); p ≤ 0.05 | NA | NA | - Statistical reduction after I |
|  | Roy et al. 2010 (65) | *∆* *I : (*reduction) | NA | NA | - Statistical reduction after I |
|  | Teodorczyk-Injeyan et al. 2021 (66) | *∆* *I : (*reduction); p = 0.01 | NA | NA | - Statistical reduction after I |
|  | | | | | |
| **IL-8**  *–*  *Proinflammatory:*  *reduction*  *expected* | Licciardone et al. 2012 (60) | *∆* *I :* -0.08 [IQR: 3.50]  *∆ C :* -0.08 [IQR: 5.13] | NR | p = 0.82 | - No statistically significant change difference between I and C |
|  | Cheng et al. 2015 (64) | *∆* *I : (*reduction); p ≤ 0.05 | NA | NA | - Statistical reduction after I |
|  | | | | | |
| **IL-10**  *–*  *Anti-inflammatory:*  *increase expected* | Bablis et al. 2022 (59) | *∆* *I :* -1.22 (statistical significant change)  *∆ C :* -0.16 (no significant change) | 1.25 [0.56, 1.93] p ≤ 0.001 | (p ≤ 0.001) | - Statistically significant decrease in I but not in C (no clinically significant, and an increase was expected) |
|  | Licciardone et al. 2012 (60) | *∆* *I :* 0.00 (IQR: 0.86)  *∆ C :* -0.04 (IQR: 0.86) | NR | P = 0.68 | - No statistically significant change difference between I and C |
|  | Lin et al. 2015 (55) | *∆* *I :* -2.28 (SD: 7.22)  *∆ C :* -1.38 (SD: 7.07) | -0.90 (SD: 7.15) | p = 0.63 | - No statistically significant change difference between I and C |
|  | Yeh et al. 2014 (57) | *∆* *I :* -2.00 (IQR: 6); p=0,06  *∆ C :* 1.00 (IQR: 11); p=0,91 | p = 0.94 | NR | - No statistically significant change difference between groups (or after treatments) |
|  | Yücesoy et al. 2021 (62) | *∆* *I :* significant increase  *∆* C *:* no significant increase | NR | NR | - Statistically significant increase after I |
|  | Teodorczyk-Injeyan et al. 2021 (66) | *∆* *I :* not significant change | NA | NA | - No statistical significance change after I |
|  | | | | | |
| **IFNγ (pg/mL)**  *–*  *Proinflammatory:*  *reduction expected* | Zgierska et al. 2016 (58) | NR | -2.1 (-8.0; 3.7) | p = 0.72 | - No statistically significant change difference between I and C |
|  | Cheng et al. 2015 (64) | *∆* *I :* reduction p ≤ 0.05 | NA | NA | - Reduction after I |
|  | Teodorczyk-Injeyan et al. 2021 (66) | *∆* *I :* not significant change | NA | NA | - No statistical significance changes after I |
| IFNγ induced Protein - IP-10 (pg/ml) | Cheng et al. 2015 (64) | *∆* *I :* reduction p ≤ 0.05 | NA | NA | - Reduction after I |
|  | | | | | |
| **CRP**  *–*  *reduction expected* | Bablis et al. 2022 (59) | I: -2.39 (significant but no clinically change)  C: -0.30 (no significant change) | 2.18 [0.84, 3.51] p ≤ 0.001 | (p ≤ 0.001) | - Decrease in I (statistical) but not in C |
|  | Nambi et al. 2020 (61) | *∆* *I*_1_ *; ∆* *I*_2_ *; ∆* *C :* (decrease) | NV | NR | - Results not reliable, inconsistency in all the article |
|  | Zgierska et al. 2016 (58) | NR | 0.8 (-0.4; 2.1) | p = 0.22 | - No statistically significant change difference between I and C |
|  | Cho et al. 2015 (63) | *∆* *I :* 0.03 [SD: 0.23]  *∆ C :* -0.1 [SD: 0.7] | NR | p > 0.05 | - No statistically significant change difference between I and C (or after treatments)   (Intention to treat analysis) |
|  | Roy et al. 2010 (65) | *∆* *I :* reduction | NA | NA | - Reduction after I |
|  | | | | | |
| **CCL2** , | Teodorczyk-Injeyan et al. 2018 (40) | I: reduction p=0.099 | NA | NA | - No statistical significance reduction after I |
| **CCL3** , | Teodorczyk-Injeyan et al. 2018 (40) | I: reduction p=0.001 | NA | NA | - Statistically significance reduction after I |
| **CCL4**  *–*  *Chemokines: reduction*  *expected* | Teodorczyk-Injeyan et al. 2018 (40) | I: reduction p ≥ 0.05 | NA | NA | - No statistical significance reduction after I |
| TA: Timepoint direct after Intervention; T_0_ : Baseline timepoint, before Intervention; C: Control group; I: Intervention group; ∆ : Differences within the group (T_A_ – T_0_); pg/mL : Concentration in picograms pro milliliter; 95%CI: 95% confidence interval; p: p-value; IQR: Interquartil range; SD: Standard Deviation; U: Mann-Whitney *U Test;* Me: Median value extracted from a graphic; NR: Not reported; NA: Do not apply; NV: Not valid, result not reliable | | | | | |
| **Sup.Table 1. Quantitative results included studies, pro biomarker** | | | | | |
|  | | **Results** | | | |
| **Biomarker** | **Article** | **Quantitative comparisons** | | | **Qualitative conclusions** |
|  |  | **within** (T_A_ – T_0_) | **between, at T**_A_ (C – I) | **time*group** *(∆ I - ∆ C)* |  |
| **TNF-α (pg/mL)**  *–*  *Proinflammatory:*  *reduction expected* | Bablis et al. 2022  (59) | *∆* *I :* -0.64 (clinically significant change)  *∆ C :* 0.22 (no significant change) | 0.87 [95%CI: 0.68, 1.06]; p ≤ 0.001 | (p ≤ 0.001) | - Clinically significant decrease in I but not in C |
|  | Licciardone et al. 2012 (60) | *∆* *I :* -1.06 [IQR: 2.11]  *∆ C :* -0.06 [IQR: 1.90] | NR | p = 0.03 | - Statistical significantly greater reduction in I than in C |
|  | Lin et al. 2015 (55) | *∆* *I :* 2.55 [SD: 24.39]  *∆ C :* -5.61 [SD: 22.96] | NR | 8.16 [SD: 23.71]; p = 0.20 | - No statistically significant change difference between I and C |
|  | Nambi et al. 2020 (76) | *∆* *I*_1_ *; ∆* *I*_2_ *; ∆* *C :* (increase) | NV | NR | - Results not reliable, inconsistency in all the article |
|  | Poojari et al. 2022 (56) | *∆* *I :* p = 0.004  *∆ C :* p = 0.002 | NR | p = 0.77 | - No statistically significant reduction difference between I and C |
|  | Yeh et al. 2014 (57) | *∆* *I :* 2.00 [IQR: 7.5]; p=0,69  *∆ C :* -5.00 [IQR: 11]; p=0,50 | p = 0.88 | NA | - No statistically significant change difference between I and C (or after treatments) |
|  | Zgierska et al. 2016 (58) | NR | 3.3 [95%CI: -2.5, 9.2] | p = 0.40 | - No statistically significant change difference between I and C |
|  | Cho et al. 2015 (61) | *∆* *I :* -0.1 [SD: 0.5]  (not significant difference)  *∆ C :* 1.4 [SD: 1.8] | NR | p < 0.01 | - Statistical significantly greater reduction in I than in C   (Intention to treat analysis) |
|  | Cheng et al. 2015 (62) | *∆* *I : (*reduction); p ≤ 0.05 | NA | NA | - Statistical reduction after I |
|  | Teodorczyk-Injeyan et al. 2021 (79) | *∆* *I :* (not significant difference) | NA | NA | - No statistically significant reduction after I |
|  | | | | | |
| **IL-1**  **(pg/mL)**  *–*  *Proinflammatory:*  *reduction expected* | Bablis et al. 2022 (59) | *∆* *I :* -1.07 (statistical significant change)  *∆ C :* 0.26 (no significant change) | 1.32 [95%CI: 0.84, 1.79]; p ≤ 0.001 | (p ≤ 0.001) | - Statistically significant decrease in I but not in C (no clinically significant) |
| **IL-1β (pg/mL)** | Licciardone et al. 2012 (60) | *∆* *I :* 0.00 [IQR: 0.00]  *∆ C :* 0.00 [IQR: 0.06] | NR | p > 0.05 | - No statistically significant change difference between I and C |
|  | Lin et al. 2015 (55) | *∆* *I :* -1.33 [SD: 5.00]  *∆ C :* -0.39 [SD: 2.09] | 0.94 [SD: 3.88] | p = 0.35 | - No statistically significant change difference between I and C |
|  | Yeh et al. 2014 (57) | *∆* *I :* 0.00 [IQR: 18]; p=0,55  *∆ C :* 0.00 [IQR: 1.5]; p=0,56 | p = 0.82 | NA | - No statistically significant change difference between I and C (or after treatments) |
|  | Zgierska et al. 2016 (58) | NR | -0.04 [95%CI: -0.6, 0.5] | p = 0.196 | - No statistically significant change difference between I and C |
|  | Cheng et al. 2015 (62) | *∆* *I :* (reduction); p ≤ 0.05 | NA | NA | - Statistical reduction after I |
|  | Teodorczyk-Injeyan et al. 2021 (79) | *∆* *I :* (not significant difference) | NA | NA | - No statistically significant reduction after I |
|  | | | | | |
| **IL-2**  *–*  *Proinflammatory:*  *reduction expected* | Lin et al. 2015 (55) | *∆* *I :* -1.24 [SD: 6.51]  *∆ C :* -0.39 [SD: 5.34] | 0.85 [SD: 5.98] | p = 0.58 | - No statistically significant change difference between I and C |
|  | Nambi et al. 2020 (76) | *∆* *I*_1_ *; ∆* *I*_2_ *; ∆* *C :* (increase) | NV | NR | - Results not reliable, inconsistency in all the article |
|  | Yeh et al. 2014 (57) | *∆* *I :* 0.00 [IQR: 5]; p=0,55  *∆ C :* 0.00 [IQR: 1.5]; p=0,59 | p = 1.00 | NA | - No statistically significant change difference between I and C (or after treatments) |
|  | Teodorczyk-Injeyan et al. 2021 (79) | *∆* *I :* (not significant difference) | NA | NA | - No statistically significant reduction after I |
|  | | | | | |
| **IL-4**  *–*  *Anti-inflammatory:*  *increase expected* | Lin et al. 2015 (55) | *∆* *I :* 0.40 [SD: 2.46]  *∆ C :* -0.93 [SD: 2.52] | 1.33 [SD: 2.49] | p = 0.05 | - Statistical significantly greater increase in I than in C (but exactly p = 0,05 and SD; between at T_A_, cross the zero) |
|  | Nambi et al. 2020 (76) | *∆* *I*_1_ *; ∆* *I*_2_ *; ∆* *C :* (increase) | NV | NR | - Results not reliable, inconsistency in all the article |
|  | Yeh et al. 2014 (57) | *∆* *I :* 0.00 [IQR: 1]; p=0,89  *∆ C :* 0.00 [IQR: 2]; p=0,66 | p = 0.94 | NA | - No statistically significant change difference between I and C (or after treatments) |
|  | | | | | |
| **IL-6 (pg/mL)**  *–*  *Proinflammatory: reduction expected* | Bablis et al. 2022 (59) | *∆* *I :* -2.05 (statistical significant change)  *∆ C :* 0.14 (no significant change) | 2.25 [95%CI: 1.32, 3.18]; p ≤ 0.001 | (p ≤ 0.001) | - Statistically significant decrease in I but not in C (no clinically significant) |
|  | Licciardone et al. 2012 (60) | *∆* *I :* 0.03 [IQR: 1.43]  *∆ C :* 0.03 [IQR: 0.47] | NR | p = 0.68 | - No statistically significant change difference between I and C |
|  | Lin et al. 2015 (55) | *∆* *I :* -2.18 [SD: 9.68]  *∆ C :* -1.28 [SD: 16.19] | NR | -0.90 [SD: 13.58]; p = 0.80 | - No statistically significant change difference between I and C |
|  | Nambi et al. 2020 (76) | *∆* *I*_1_ *; ∆* *I*_2_ *; ∆* *C :* (increase) | NV | NR | - Results not reliable, inconsistency in all the article |
|  | Yeh et al. 2014 (57) | *∆* *I :* -0.50 [IQR: 9]; p=0,95  *∆ C :* 0.00 [IQR: 7.5]; p=0,81 | p = 0.70 | NA | - No statistically significant change difference between I and C (or after treatments) |
|  | Yücesoy et al. 2021 (66) | *∆* *I :* (reduction but not significant)  *∆* C *:* (reduction but not significant) | NR | NR | - No statistically significant reduction after I |
|  | Zgierska et al. 2016 (58) | NR | 0.2 [95%CI: -1.3, 1.8] | p = 0.126 | - No statistically significant change difference between I and C |
|  | Cheng et al. 2015 (62) | *∆* *I : (*reduction); p ≤ 0.05 | NA | NA | - Statistical reduction after I |
|  | Roy et al. 2010 (71) | *∆* *I : (*reduction) | NA | NA | - Statistical reduction after I |
|  | Teodorczyk-Injeyan et al. 2021 (79) | *∆* *I : (*reduction); p = 0.01 | NA | NA | - Statistical reduction after I |
|  | | | | | |
| **IL-8**  *–*  *Proinflammatory:*  *reduction*  *expected* | Licciardone et al. 2012 (60) | *∆* *I :* -0.08 [IQR: 3.50]  *∆ C :* -0.08 [IQR: 5.13] | NR | p = 0.82 | - No statistically significant change difference between I and C |
|  | Cheng et al. 2015 (62) | *∆* *I : (*reduction); p ≤ 0.05 | NA | NA | - Statistical reduction after I |
|  | | | | | |
| **IL-10**  *–*  *Anti-inflammatory:*  *increase expected* | Bablis et al. 2022 (59) | *∆* *I :* -1.22 (statistical significant change)  *∆ C :* -0.16 (no significant change) | 1.25 [0.56, 1.93] p ≤ 0.001 | (p ≤ 0.001) | - Statistically significant decrease in I but not in C (no clinically significant, and an increase was expected) |
|  | Licciardone et al. 2012 (60) | *∆* *I :* 0.00 (IQR: 0.86)  *∆ C :* -0.04 (IQR: 0.86) | NR | P = 0.68 | - No statistically significant change difference between I and C |
|  | Lin et al. 2015 (55) | *∆* *I :* -2.28 (SD: 7.22)  *∆ C :* -1.38 (SD: 7.07) | -0.90 (SD: 7.15) | p = 0.63 | - No statistically significant change difference between I and C |
|  | Yeh et al. 2014 (57) | *∆* *I :* -2.00 (IQR: 6); p=0,06  *∆ C :* 1.00 (IQR: 11); p=0,91 | p = 0.94 | NR | - No statistically significant change difference between groups (or after treatments) |
|  | Yücesoy et al. 2021 (66) | *∆* *I :* significant increase  *∆* C *:* no significant increase | NR | NR | - Statistically significant increase after I |
|  | Teodorczyk-Injeyan et al. 2021 (79) | *∆* *I :* not significant change | NA | NA | - No statistical significance change after I |
|  | | | | | |
| **IFNγ (pg/mL)**  *–*  *Proinflammatory:*  *reduction expected* | Zgierska et al. 2016 (58) | NR | -2.1 (-8.0; 3.7) | p = 0.72 | - No statistically significant change difference between I and C |
|  | Cheng et al. 2015 (62) | *∆* *I :* reduction p ≤ 0.05 | NA | NA | - Reduction after I |
|  | Teodorczyk-Injeyan et al. 2021 (79) | *∆* *I :* not significant change | NA | NA | - No statistical significance changes after I |
| IFNγ induced Protein - IP-10 (pg/ml) | Cheng et al. 2015 (62) | *∆* *I :* reduction p ≤ 0.05 | NA | NA | - Reduction after I |
|  | | | | | |
| **CRP**  *–*  *reduction expected* | Bablis et al. 2022 (59) | I: -2.39 (significant but no clinically change)  C: -0.30 (no significant change) | 2.18 [0.84, 3.51] p ≤ 0.001 | (p ≤ 0.001) | - Decrease in I (statistical) but not in C |
|  | Nambi et al. 2020 (76) | *∆* *I*_1_ *; ∆* *I*_2_ *; ∆* *C :* (decrease) | NV | NR | - Results not reliable, inconsistency in all the article |
|  | Zgierska et al. 2016 (58) | NR | 0.8 (-0.4; 2.1) | p = 0.22 | - No statistically significant change difference between I and C |
|  | Cho et al. 2015 (61) | *∆* *I :* 0.03 [SD: 0.23]  *∆ C :* -0.1 [SD: 0.7] | NR | p > 0.05 | - No statistically significant change difference between I and C (or after treatments)   (Intention to treat analysis) |
|  | Roy et al. 2010 (71) | *∆* *I :* reduction | NA | NA | - Reduction after I |
|  | | | | | |
| **CCL2** , | Teodorczyk-Injeyan et al. 2018 (40) | I: reduction p=0.099 | NA | NA | - No statistical significance reduction after I |
| **CCL3** , | Teodorczyk-Injeyan et al. 2018 (40) | I: reduction p=0.001 | NA | NA | - Statistically significance reduction after I |
| **CCL4**  *–*  *Chemokines: reduction*  *expected* | Teodorczyk-Injeyan et al. 2018 (40) | I: reduction p ≥ 0.05 | NA | NA | - No statistical significance reduction after I |
| TA: Timepoint direct after Intervention; T_0_ : Baseline timepoint, before Intervention; C: Control group; I: Intervention group; ∆ : Differences within the group (T_A_ – T_0_); pg/mL : Concentration in picograms pro milliliter; 95%CI: 95% confidence interval; p: p-value; IQR: Interquartil range; SD: Standard Deviation; U: Mann-Whitney *U Test;* Me: Median value extracted from a graphic; NR: Not reported; NA: Do not apply; NV: Not valid, result not reliable | | | | | |
